# Anionic Copolymerization of Styrene Sulfide with Elemental Sulfur (S_8_)

**DOI:** 10.3390/ma13112597

**Published:** 2020-06-07

**Authors:** Jakub Wręczycki, Dariusz M. Bieliński, Marcin Kozanecki, Paulina Maczugowska, Grzegorz Mlostoń

**Affiliations:** 1 Institute of Polymer and Dye Technology, Faculty of Chemistry, Lodz University of Technology, 12/16 Stefanowskiego Street, 90-924 Lodz, Poland; dariusz.bielinski@p.lodz.pl; 2 Department of Molecular Physics, Faculty of Chemistry, Lodz University of Technology, 116 Zeromskiego Street, 90-924 Lodz, Poland; marcin.kozanecki@p.lodz.pl (M.K.); paulina.maczugowska@dokt.p.lodz.pl (P.M.); 3 Department of Organic and Applied Chemistry, Faculty of Chemistry, University of Lodz, 12 Tamka Street, 91-403 Lodz, Poland; grzegorz.mloston@chemia.uni.lodz.pl

**Keywords:** styrene sulfide, poly (styrene sulfide), polysulfides, elemental sulfur copolymers

## Abstract

The superior ability of thiiranes (episulfides) to undergo ring-opening polymerization (ROP) in the presence of anionic initiators allows the preparation of chemically stable polysulfide homopolymers. Incorporation of elemental sulfur (S_8_) by copolymerization below the floor temperature of S_8_ permits the placement of a large quantity of sulfur atoms in the polysulfide mainchain. The utility of styrene sulfide (2-phenylthiirane; StS) for copolymerization with elemental sulfur is reported here. A few polysulfides differing depending on the initial ratio of S_8_ to StS and copolymerization time were synthesized. Various spectroscopic methods (^1^H NMR, ^13^C NMR, Raman spectroscopy and FTIR spectroscopy) were applied to characterize the chemical structure of the copolymers. Additionally, the phase structure and thermal stability of the synthesized polysulfides were investigated using DSC and TGA, respectively. The successful anionic copolymerization of styrene sulfide and elemental sulfur has been demonstrated.

## 1. Introduction

The preparation of polymeric materials with novel structures and simultaneously interesting and preferably smart properties has a big priority in modern polymer chemistry [1,2]. Modification of end- and side-groups or mainchain architecture is a common way to tailor the properties of polymeric materials. However, such modifications do not always provide materials with properties obtainable, for example, by the incorporation of heteroatoms into the polymer mainchain [3]. The most quintessential examples are polyethers, which have found widespread industrial implementation as epoxy resins due to their unique and useful properties [4]. Incorporation of some elements of the p-block such as sulfur [5,6], boron [5,7] or prominently phosphorous [3,5] into the carbon mainchain permits the formation of more exotic structures that should be of importance for polymer synthesis development. The biggest limitation is mostly the lack of effective synthetic methods for the incorporation of these elements into long polymeric chains. For the larger than academic scale production, monomer availability and consequent costs must also be considered.

Polymers with sulfur atoms attached to alkylene or arylene groups (carbon mainchain) via sigma bonds are widely known as polysulfides with general formula [RS_x_]_n_. Depending on the sulfur rank (x-number of sulfur atoms incorporated into the mainchain), polysulfides may be divided into poly (monosulfides) (x = 1) (sulfur analogues of polyethers) and poly (polysulfides) (x ≥ 2) (lack of oxygen analogues) [8]. To date, the most frequently used methodology for their synthesis is a polycondensation reaction of dihaloalkanes or dihaloarenes with sodium sulfide. This method has been employed for the synthesis of the poly (phenylene sulfide) (PPS), undebatable the flagship example of polysulfide polymers. This material was first synthesized by Macallum [9,10,11], then on an industrial scale by Edmonds and Hill from the Phillips Petroleum Co. (U.S. Patent 3,354,129) [12]. Other important methodologies leading to polysulfide polymers include polyaddition of thiols to olefins and ring-opening polymerization (ROP) of cyclic sulfides of different ring size and number of sulfur atoms [8].

The ability of thiiranes (episulfides) to undergo ring-opening polymerization is well recognized in polysulfide synthesis. Various three-membered sulfur heterocycles have been successfully polymerized in the past [13,14]. However, not enough attention has been focused on the polymerization of styrene sulfide (2-phenylthiirane), the simplest episulfide functionalized with an aromatic ring. The very first report concerning its polymerization appeared in 1961 [15]. Poly (styrene sulfide) was prepared via bulk polymerization in the presence of different catalysts, leading to a white, fluffy polymeric product. Styrene sulfide has also been polymerized electrochemically (anodically and cathodically) in organic media (e.g., acetonitrile) using different materials as electrodes to obtain white powdery polymers [16]. Taking into account the structure of thiiranes and its analogy to that of epoxide derivatives, copolymerization potential would seem to be of interest. One approach is the copolymerization with elemental sulfur (S_8_) aimed at incorporation of multiple sulfur atoms into the polysulfide mainchain.

An efficient method for thiirane copolymerization with elemental sulfur leading to chemically stable poly (polysulfides) has been proposed [17,18]. This process allows the generation of high molecular weight compounds (M_n_~10^4^) in very good yields. Products contained up to 85 wt% chemically bonded sulfur atoms in the structure and exhibited elastomeric properties. The high efficiency of anionic copolymerization of thiiranes with S_8_ results from the similarity of the active centers derived from the cyclooctasulfur and thiirane that form the polythiolate anions (~S_x_^−^). Under controlled conditions (lack of water, oxygen, and electrophilic impurities), unfavorable chain termination reactions are limited, and the process becomes a “living” one [19,20,21].

**Figure 1:**
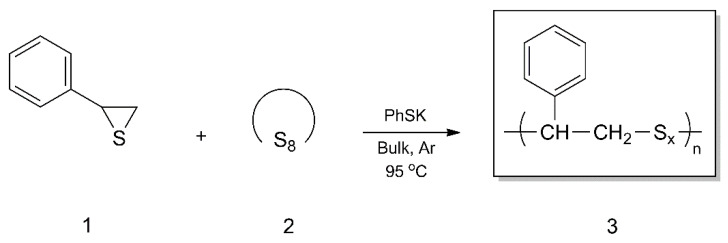
Copolymerization of styrene sulfide (**1**) with elemental sulfur (S_8_).

The propensity for styrene sulfide (**1**) to undergo copolymerization with elemental sulfur (S_8_) **2**, below the thermodynamic barrier of sulfur homopolymerization (the floor temperature), using potassium thiophenolate (PhSK) as an anionic initiator (Figure 1) is described here for the first time. The impact of the initial monomer ratio was investigated in terms of the sulfur rank (x) and consequent structure and thermal properties of synthesized polysulfides **3**. Different spectroscopic methods were applied to characterize the structure of the obtained copolymers (^1^H NMR, ^13^C NMR, Raman and FTIR spectroscopy) and to demonstrate that a true copolymerization process had been acquired. In addition, DSC and TGA analyses were performed to indicate the phase structure and the thermal stability of the synthesized polysulfides.

## 2. Materials and Methods

### 2.1. Materials

Racemic (+/−)-styrene oxide (StO) (98%) and potassium thiophenolate (100%) were purchased from abcr (Karlsruhe, Germany). Thiourea (99%) was provided by Carl Roth (Karlsruhe, Germany). Molecular sieves 4Å (mesh 8–12) and elemental sulfur (S_8_) in the form of powder (99.98%) were purchased from Merck (Sigma-Aldrich, Darmstadt, Germany). Allyl bromide (99%) was provided by Alfa Aesar (Haverhill, MA, USA). Organic solvents and analytical compounds were received from the following companies: methanol (99.8%), diethyl ether (99.5%), dichloromethane (99.5%) from Chempur (Piekary Śląskie, Poland), chloroform-d (99.8%) (CDCl_3_) from Armar Chemicals (Döttingen, Switzerland), styrene (99%) from Acros Organics (Geel, Belgium). The reference poly *(*styrene) (PSt) was synthesized in our laboratory via suspension polymerization.

### 2.2. General Synthesis Procedure of the Styrene Sulfide (StS)

Styrene sulfide (StS) was obtained according to the methodology previously reported [22]. Typically, 1.20 g of styrene oxide (10 mmol), 1.52 g of thiourea (20 mmol) and 1.0 g of molecular sieves 4 Å were placed in a round-bottomed flask (50 mL) equipped with a magnetic stirrer and overflown with 30 mL of methanol. The resulting mixture was heated to reflux under condenser and stirred in boiling methanol for 45–60 min. After approximately 15 min, the reaction mixture started to yield a milky (cloudy) suspension. At the end, the methanol was precisely evaporated, and the reaction mixture was overflown with 15 mL of diethyl ether, then stirred again for 10 min. The final mixture was filtered, and the isolated solid was washed three times with a portion of 5 mL of diethyl ether each time. The organic solvent was evaporated to give a slightly-yellow, water-like viscous liquid in 53–64% yield. In the anionic polymerization, purity of reagents is crucial to retain its “living” character. Column chromatography has been applied to purify StS before the (co) polymerization experiment. However, considerable degradation to styrene and elemental sulfur (S_8_) prompted us to use styrene sulfide without additional purification. Starting StS was conditioned in a closed vial at room temperature and (co) polymerized each time no later than 24 h after its synthesis.

### 2.3. General Procedure for the Synthesis of Poly (Styrene Sulfide) (PStS) and Poly (StS/S_8_) Copolymers

In a two-necked round-bottom flask (10 mL), elemental sulfur (0–3 equiv. “S_1_”), potassium thiophenolate (PhSK) (0.0019 equiv.) and styrene sulfide (StS) (1 equiv.) were placed in the presented order. The reaction flask was equipped with a magnetic stirrer, a condenser endowed in a tube with calcium chloride (CaCl_2_) and a custom airtight degassing system. The degassing system consisted of a small cylindrical dropping funnel, closed on the top with a rubber septum equipped with argon-filled balloon applied by a syringe, connected to the vacuum receiving tube (105°) placed in the second neck of the reaction flask and closed with the empty balloon covered with a parafilm. The (co) polymerization mixtures were degassed before heating and stirring by applying argon via the degassing system (turn on a tap in the dropping funnel) and oxygen presented in the flask was collected in the empty balloon. After successful degassing, the solution was placed in an oil bath, heated to 95 °C and stirred for the appropriate time. Then, to the very viscous mixture or solid, a few droplets of allyl bromide (chain termination agent) solution in CH_2_Cl_2_ were added to dissolve the (co) polymerization product. The dissolved mixture was stirred for a few minutes and then the (co) polymer was precipitated in cold methanol, filtered, and dried for at least 24 h at 25 °C in a laboratory vacuum oven. Each polysulfide was at least two times recrystallized from cold methanol in order to remove residual monomer. The detailed synthesis conditions are described and discussed in Section 3.2.1.

### 2.4. Raman Spectroscopy Analysis

The Raman spectroscopy studies were performed at room temperature using a dispersive spectrometer Jobin-Yvon T-64000 (Horiba, Ltd., Kyoto, Japan) equipped with confocal microscope Olympus BX-40 (Olympus Co., Tokyo, Japan). The Ar laser line (wavelength 514.5 nm, power at the sample surface ca. 5 mW) was used as a source of excitation. The acquisition time was 30 s and two scans were averaged. The measurements were carried out on the solid samples in form of powders (polymers) and liquids (monomers and other analytes).

### 2.5. FTIR Spectroscopy Analysis

The Fourier transform infrared (FTIR) spectroscopy studies were performed at room temperature by using a Thermo Scientific Nicolet 6700 FTIR spectrometer (Thermo Fisher Scientific, Waltham, MA, USA) equipped with diamond Smart Orbit ATR sampling accessory. The FTIR absorbance spectra were investigated in the absorption mode (64 scans) within the wavenumber range of 4000–400 cm^−1^ with the spectral resolution set to 4 cm^−1^.

### 2.6. DFT Calculations

The optimization of particle geometry and calculation of infrared absorption and Raman spectra for various model poly (styrene sulfides) was performed in the Gaussian 09W software [23]. Calculations were carried out using the B3LYP [24,25,26,27]/6-31G (d,p) [28,29] method. The obtained spectra were corrected using scaling factor 0.961. Four various model poly (styrene sulfides) differing in the length of S_x_ bridges (x = 1–4) were studied. Due to limited computational resources, five-monomer-units oligomers were used in the calculations.

### 2.7. ^1^H NMR and ^13^C NMR Spectroscopy Analysis

The nuclear magnetic resonance (NMR) spectra were measured using a Bruker Avance III apparatus (Bruker, Billerica, MA, USA) at 600 MHz (^1^H NMR) and at 150 MHz (^13^C NMR), respectively. The measurements were carried out in d-chloroform (CDCl_3_), using solvent signals as a reference (^1^H NMR, δ = 7.26 ppm (CDCl_3_); ^13^C NMR, δ = 77.0 ppm (CDCl_3_)). Chemical shifts (δ) were presented in ppm. The mass of the pure polysulfides samples were approximately 15 mg for ^1^H NMR and around 100 mg for ^13^C NMR measurements.

### 2.8. Thermal Analysis (TGA-DSC)

The thermogravimetric analysis (TGA) and differential scanning calorimetry (DSC) analysis were carried out by using a Mettler Toledo TGA/DSC 1 STARe System (Mettler Toledo, Greifensee, Switzerland) equipped with a Gas Controller GC10 (Mettler Toledo, Greifensee, Switzerland). The TGA tests were performed in a temperature range from 25 °C to 900 °C (25–500 °C under argon atmosphere, and next 500–900 °C under air atmosphere) with a heating rate of 10 °C/min. The DSC samples were firstly cooled from room temperature to −80 °C with a cooling rate of 10 °C/min and then heated and investigated in a temperature range from −80 °C to 200 °C with a heating rate of 10 °C/min. Both tests were carried out on a small powder samples (6–7 mg).

## 3. Results and Discussion

### 3.1. Analysis of the Styrene Sulfide (StS)

The analysis of the monomer (styrene sulfide) was carried out in order to confirm its structure before the (co) polymerization process and assure that styrene (St), which is a product of StS degradation, is not present in the StS. Styrene sulfide stored at low temperature may eliminate the sulfur atom furnishing styrene (St) and elemental sulfur (S_8_) or undergoes autopolymerization at room temperature in the absence of added initiator [15]. Moreover, the current knowledge of styrene sulfide is very poor, especially in terms of spectroscopic data; hence, it is advisable to supplement the information about spectroscopic properties of StS, and two complementary methods, i.e., Raman and FTIR spectroscopy, were applied. Since the bonds in the structure of styrene sulfide exhibit rather non-polar character (C–S and C–C), the Raman spectroscopy seems to be a perfect analytical tool for both, the monomer and polysulfide polymers analysis.

#### 3.1.1. Raman Spectroscopy Analysis

**Figure 2:**
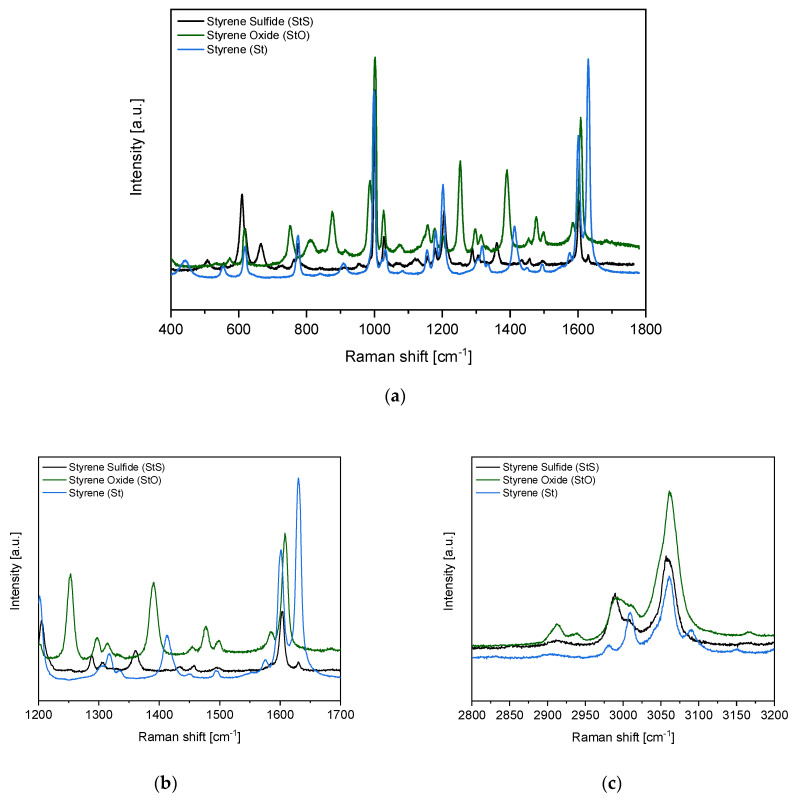
Raman spectrum of styrene oxide (StO)—substrate, and styrene sulfide (StS)—product (monomer), (**a**) in the middle Raman shift region; (**b**) in the Raman shift region of significant changes; (**c**) in the high Raman shift region. The Raman spectrum of styrene (St) was added for comparison.

The Raman spectra of styrene sulfide (monomer), styrene oxide and styrene in the 400–1800 cm^−1^ region as well as 2800–3200 cm^−1^ are shown in Figure 2.

Comparing the StS and StO Raman spectra presented in Figure 2a, one can notice that in the styrene sulfide spectrum a new band centered at 665 cm^−1^ appeared, which is not observed in the styrene oxide spectrum. This band can be assigned to the stretching vibration of the C–S bond in the thiirane ring [30]. It constitutes the main evidence of the successful synthesis of styrene sulfide and its structure confirmation. Moreover, the successful conversion of styrene oxide into styrene sulfide is confirmed by a lack of the characteristic epoxy ring breathing band in the StS spectrum, which is visible well in the StO spectrum at 1252 cm^−1^ [31]. Additionally, in Figure 2b, the characteristic absorption bands in the spectrum of styrene at 1630 cm^−1^ assigned to the vinyl ν (C=C) and at 1414 cm^−1^ corresponding to the vinyl=CH_2_ group [32,33] are not present in the spectrum of styrene sulfide. Comparing the high Raman region (Figure 2c), one can see that the band related to vinyl stretch at 3010 cm^−1^ is also not present in the spectrum of styrene sulfide. It suggests the fairly good stability of the StS and excludes its degradation to styrene and S_8_ at room temperature in a time period of up to 24 h. The mutual bands characteristic of the aromatic ring both in StS and St are overlapping, for example the bands at ca. 620 cm^−1^ in St and slightly shifted in the case of StS at ca. 610 cm^−1^ (ring deformation mode), at 1000 cm^−1^ (ring breathing mode) and ca. 1600 cm^−1^ (ring skeletal stretch). Moreover, the bands assigned to the C−H bond, at 775 cm^−1^ (C–H out of plane deformation) and at 1030 cm^−1^ (C–H in plane deformation) are also overlapping [33].

#### 3.1.2. FTIR Spectroscopy Analysis

**Figure 3:**
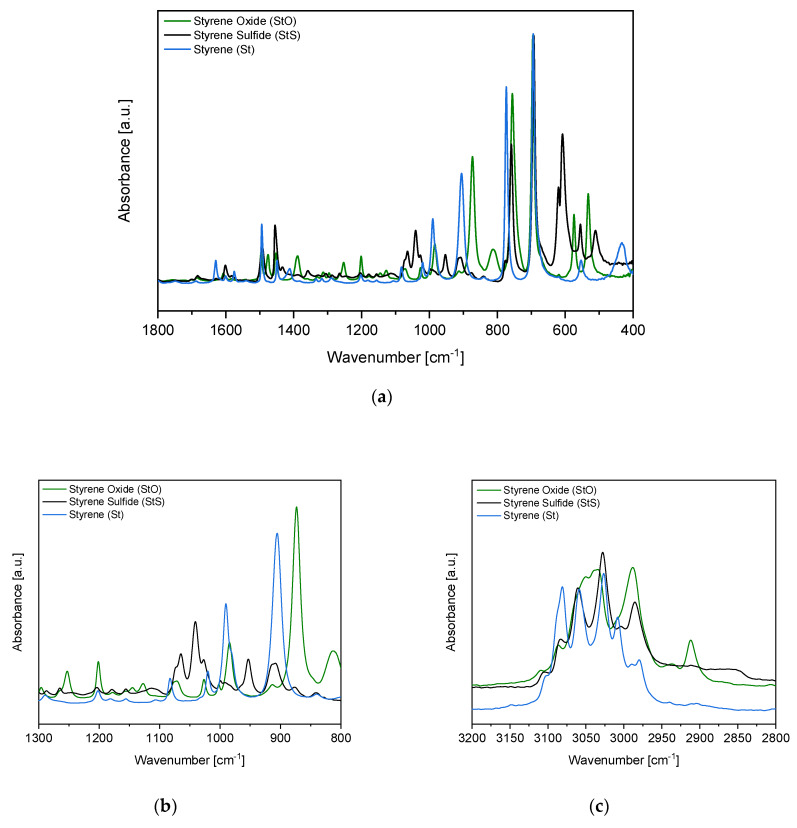
FTIR spectrum of styrene oxide (StO)—substrate, and styrene sulfide (StS)—product (monomer), (**a**) in the middle wavenumber region; (**b**) in the wavenumber region of significant changes; (**c**) in the high wavenumber region. The FTIR spectrum of styrene (St) was added for comparison.

The FTIR spectra of styrene sulfide (monomer), styrene oxide and styrene in the 400–1800 cm^−1^ region as well as 2800–3200 cm^−1^ are presented in Figure 3.

As expected, in the IR spectrum of styrene sulfide (StS) (Figure 3a), a new band in the range from 610–620 cm^−1^ appeared, which is not present in the spectra of both styrene oxide and styrene. In this spectral region, lines characteristic of the C–S bond are located, and in this case correspond to the asymmetric deformation of the thiirane ring. The band responsible for the symmetric deformation of the episulfide ring appears as well in the StS spectrum at 1041 cm^−1^ [34]. Furthermore, in the styrene oxide spectrum the characteristic bands for the deformation of the epoxide group (C‒O‒C) ca. 873 cm^−1^ as well as 1253 cm^−1^ are present, which completely disappeared and are not visible in the StS spectrum [35]. It indicates the effective conversion of the epoxide to the thiirane. Comparing the regions at 905–915, 985–995 and near 1630 cm^−1^, where bands characteristic for the vibrations of vinyl groups appear, we can unambiguously claim that in the synthesized styrene sulfide, styrene does not occur [33]. The FTIR spectra confirm the successful synthesis of the monomer (StS), used in the further reactions.

### 3.2. Analysis of of Poly (Styrene Sulfide) (Poly (StS)) and Copolymers of StS/S_8_

The analysis of poly (styrene sulfide) and the copolymers of styrene sulfide/S_8_ was performed in order to investigate the chemical structure of synthesized polysulfides and to confirm that the sulfur transfer from elemental sulfur to thiirane took place. In addition, the present discussion aims to provide the evidence that sulfur atoms are chemically bonded in the polysulfide architecture after the (co) polymerization process. We are aware of the fact, that in order to confirm the anionic character of the reaction and homogeneity of the synthesized copolymers, gel permeation chromatography (GPC) tests should be performed. Because it was not the main objective of the presented studies, we decided to rely on the results justifying the statement, derived from other experimental techniques applied. The problem will be revisited in a future study and published elsewhere.

#### 3.2.1. Synthesis Conditions of Polysulfides and Their Compositions

Poly (styrene sulfide) and three different StS/S_8_ copolymers were synthesized. The synthesis procedure was described in Section 2.3, while the (co) polymerization conditions as well as the composition of the synthesized (co) polymers are provided in Table 1.

The anionic homopolymerization of **1** and its copolymerization with elemental sulfur by using potassium thiophenolate as an initiator was performed in bulk at 95 °C. The reason for carrying out the copolymerization process at an elevated temperature was to increase the solubility of S_8_ in **1** at maximum. However, the temperature was selected carefully to not exceed the melting point of sulfur (approximately 119 °C).

The S_1 [0]_/StS _[0]_ ratio was calculated as an initial mole content of sulfur atom (“S_1_”) to styrene sulfide, while the S_8 [0]_/StS _[0]_ ratio was expressed as a mole content of elemental sulfur (S_8_) to StS. The content of sulfur in the (co) polymerization mixture expressed as a S_1 [0]_/StS _[0]_ ratio increased from 0.0 in the case of poly (StS) to 1.0, intended to obtain polysulfides with various sulfur atom quantity in the hydrocarbon repeating unit of polysulfides (sulfur rank). The reaction time was set to 2 or 4 h in order to observe the impact of the reaction time on the chemical structure (sulfur content) of synthesized polysulfides and overall physical properties.

The homopolymer of styrene sulfide is a white fluffy powder, while its copolymers with sulfur are ivory or light-yellow powders, depending on the initial sulfur content. All the studied (co) polymers are excellently soluble in chloroform (CHCl_3_), dichloromethane (CH_2_Cl_2_) and tetrahydrofuran (THF). However, they are not soluble in methanol and diethyl ether and partially soluble in toluene, if the S_1 [0]_/StS _[0]_ ratio does not exceed 0.5.

#### 3.2.2. Raman Spectroscopy Analysis

**Figure 4:**
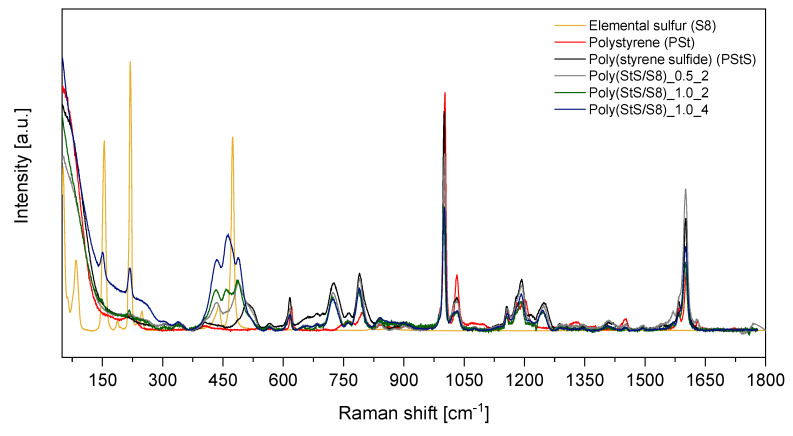
Raman spectra of synthesized polysulfides with different sulfur content. The spectra registered for elemental sulfur and reference polystyrene were added for comparison.

**Figure 5:**
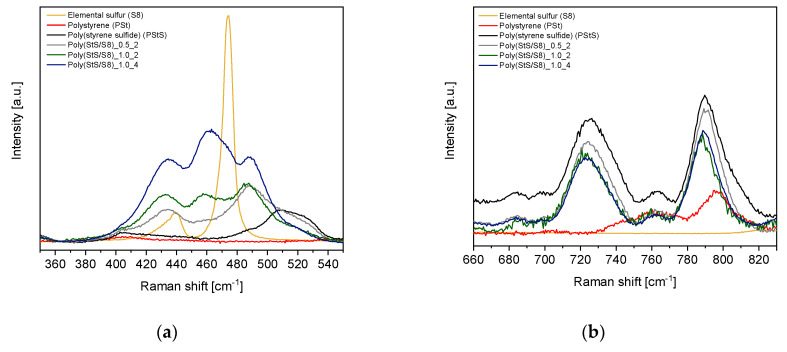
Raman spectra of synthesized polysulfides: (**a**) in the range characteristic for the polysulfide (-S_x_-) bonds, and (**b**) in the range characteristic for the C–S bonds.

**Figure 6:**
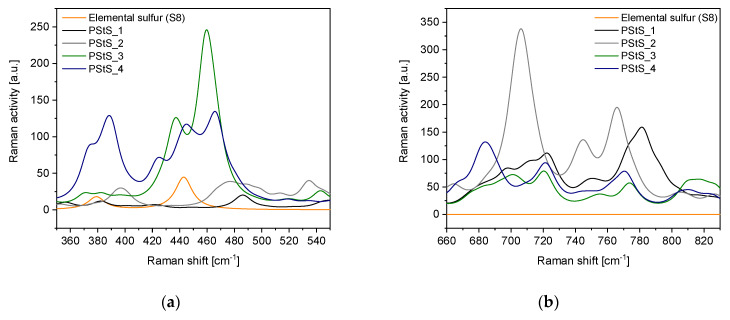
Calculated Raman spectra of poly (styrene oligosulfides) with various length of sulfur bridges: (**a**) in the range characteristic for the polysulfide (-S_x_-) bonds, (**b**) in the range characteristic for the C–S bonds. The spectrum of elemental sulfur was added for comparison.

The Raman spectra of elemental sulfur, the poly (styrene) reference and synthesized homopolymer of styrene sulfide and its copolymers with S_8_ are depicted in Figure 4. The spectral region characteristic for the stretching modes of various sulfide bonds (-S_x_-) in linear polysulfides as well as the stretching modes of carbon-sulfur (C‒S) bonds are included in Figure 5 (experimental data) and Figure 6 (calculated data).

Elemental sulfur (S_8_), as a non-polar substance, gives few very strong and characteristic Raman lines at 81, 151, 217, 248, 437 and 475 cm^−1^ [36]. The strongest ones with maxima at 151 and 217 cm^−1^ are visible also in the Raman spectra of our products, but their intensity proves that the concentration of S_8_ in obtained polysulfides is very low. Raman spectroscopy provides two significant proofs of successful anionic (co) polymerization of **1** with S_8_: (i) lack or low intensity of S_8_ bands in the synthesized poly (StS/S_8_) and simultaneously new bands which may be assigned to linear sulfur structures (S‒S bonds) in the spectral region 380 to 540 cm^−1^ (see Figure 5a) [19,21]. (ii) line corresponding to C‒S bonds visible in Raman spectra between 700–750 cm^−1^ (see Figure 5b) [21]. Detailed analysis of Raman spectra shows, however, the differences in the final content of sulfur in the polysulfide structure manifested through the various intensity of Raman lines related to S‒S bonds depending on initial S_1 [0]_/StS _[0]_ ratio.

Comparison of the poly (StS) and poly (StS/S_8_) copolymers spectra with the poly (St) spectrum enables to notice the new band or even a set of bands that do not occur in the poly (styrene). The first new peak is located at 700–750 cm^−1^ with maximum at around 725 cm^−1^ and corresponds to the stretching vibration of the C‒S bond in the linear structure of synthesized polysulfides. In every case, the band is narrow with significant intensity.

In the spectral range related to the S‒S stretching modes (380–540 cm^−1^) in linear polysulfides, a complex, multipeak band is visible. In case of the poly (styrene sulfide), the spectrum shows a single broad peak with maximum at around 510 cm^−1^. Its presence is surprising because the S‒S bond should not occur in the structure of styrene sulfide homopolymers. However, its appearance may be an evidence for the partial head-to-head configuration of macromolecules, next to the preferred head-to-tail configuration. The head-to-tail configuration results from attack of active center (-S_x_-) onto the methylene carbon atom in the thiirane ring and was confirmed in the anionic polymerization of 2-methylthiirane and 2,2-dimethylthiirane [21,37], whereas the head-to-head configuration of monomeric fragments may be arranged in the redistribution reactions of macromolecules [21]. Another explanation of its presence may be just an undesirable layering of nearby bands in vibrational spectra. The Raman spectra of poly (StS/S_8_) copolymers vary considerably in the range of 380–540 cm^−1^ comparing to the Raman spectrum of poly (StS). A broad band related to S‒S stretching modes displays discreate nature with two or three distinct peaks of variable intensity ratio, depending on initial sulfur content and (co) polymerization time. In the Raman spectrum of the poly (StS/S_8_)_0.5_2 copolymer, two peaks with maxima at 433 and 488 cm^−1^ appear, whereas for the rest of synthesized polysulfides three clearly visible peaks may be distinguished with maxima at 433, 459 and 486 cm^−1^ in the case of poly (StS/S_8_)_1.0_2 and 433, 462 and 488 cm^−1^ in the case of poly (StS/S_8_)_1.0_4. None of them may be ascribed neither to the elemental sulfur (S_8_) signals, nor to StS. The broad band located between 380 and 540 cm^−1^ (instead of distinct and separated bands) is a result of the overlapping of stretching vibration bands that correspond to the macromolecules with diversified sulfur rank.

Results of DFT studies fully confirm the above presented interpretation of the Raman spectra. The calculation was performed for poly (styrene oligosulfides) with diverse length of the sulfur bridges -S_x_- (x = 1–4), wherein system with x = 1 (PStS_1) is a model of poly (StS). It is necessary to underline that some shift between the position of individual bands as well as the difference in relative intensities in calculated and measured spectra might be expected. This is because: (i) calculations were carried out for short oligomers and therefore not all possible chain conformations were taken under consideration, (ii) calculations did not involve intermolecular interactions as a single chain was modeled, only (iii) the harmonic approximation was used in the presented calculations. Due to these factors, the scaling factor equal 0.961 was applied for better fitting the results of calculations to experimental data. This is a typical value for the applied B3LYP/6-31G (d,p) method. Considering all above presented arguments, only qualitative comparison between calculations and experiments is possible. Nevertheless, trends observed in the range of 350–550 cm^−1^ of calculated spectra suggest that the lengthening of sulfide bridges results in the appearance of a complex, multipeak band in Raman spectrum of poly (styrene oligosulfides), while for PStS_1 broad but single band shifted to higher wavenumber in comparison to elemental sulfur is observed. Analogues trends are observed in the experimental data. Comparison of calculated and experimental data in this spectral range proves that the performed synthesis with usage of elemental sulfur allowed to obtain the products which form a mixture of poly (styrene oligosulfides) with sulfur bridges of different length, with dominant contribution of polymers with sulfur atom sequence longer than 2. Similar, however not unequivocal conclusions may be formulated based on analysis of the spectral region 660–830 cm^−1^.

The characteristic for the poly (styrene) bands [32,38,39] that should also be presented in the spectrum of poly (styrene sulfide) and poly (StS/S_8_) copolymers resulting from the similarity of both structures are maintained. The bands assigned to the phenyl ring are overlapping and are located nearby 620 cm^−1^ (ring deformation mode), 1000 cm^−1^ (ring breathing mode), and 1600 cm^−1^ (ring-skeletal stretch). Furthermore, the bands related to the C‒H out-of-plane deformation and C‒H in-plane deformation are consistent as well and can be found at 795 cm^−1^ in the case of poly (styrene) and slightly shifted to 790 cm^−1^ in the case of poly (StS) and its copolymers with sulfur at ca. 1030 cm^−1^, respectively. The bands related to the C‒C stretching and CH_2_ scissors modes are also consistent and located in the regions of 1150–1200 cm^−1^, and 1450 cm^−1^, respectively in every case.

#### 3.2.3. FTIR Spectroscopy Analysis

**Figure 7:**
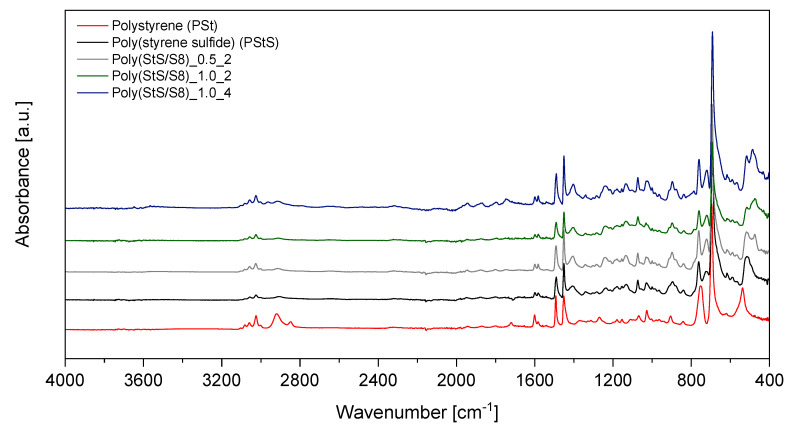
The FTIR spectra of the synthesized polysulfides and reference poly (styrene).

**Figure 8:**
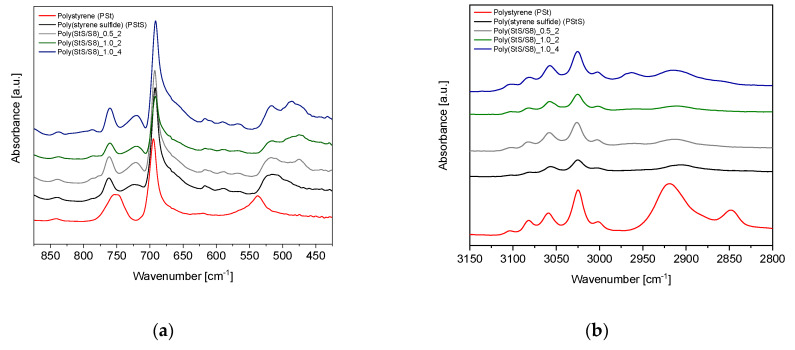
The FTIR spectra zoomed on (**a**) the spectral range characteristic for C–S and S–S stretching modes, (**b**) the spectral range including C–H stretching modes.

**Figure 9:**
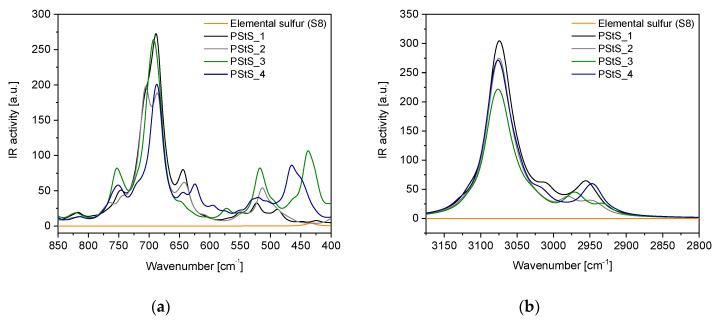
Calculated IR spectra of olisulfides with various lengths of sulfur bridges: (**a**) in the range characteristic for the polysulfide (-S_x_-) bonds, and (**b**) in the range characteristic for the C–S bonds.

The FTIR spectra of poly (styrene sulfide) and poly (StS/S_8_) copolymers as well as the reference poly (styrene) in the overall scope (4000–400 cm^−1^) are shown in Figure 7. The range of measurement characteristic for substantial bonds is shown in Figure 8 (experimental data) and Figure 9 (calculated data).

Comparing the absorption FTIR spectra of poly (styrene sulfide) and poly (StS/S_8_) copolymers with poly (styrene), it is easy to detect the appearance of the new peak at around 720 cm^−1^, which may be attributed to the C‒S bond in the linear structure of polysulfides [40]. This band occurs also in the FTIR spectra of all other polysulfides. Moreover, in case of poly (StS) and its copolymers with sulfur, the range below 540 cm^−1^ is rich in overlapped lines, due to the occurrence of diversified types of sulfide bonds in the macromolecules. Depending on the initial ratio of styrene sulfide to elemental sulfur and the copolymerization time, the number of sulfur atoms in the mer structure of synthesized polysulfides is different. This fact is confirmed by the various shapes, the number of maxima in the rage of 450–540 cm^−1^ and their relative intensity ratio. However, due to the fact that the sulfide bonds possess strong non-polar character, the changes are much clearly visible in the Raman spectra in the range of 380–540 cm^−1^ as described in Section 3.2.2. In the case of poly (StS) and poly (StS/S_8_) copolymers, residual peaks along the particular spectrum are overlapping suggesting that the polysulfide macromolecules differ only in the number of sulfur atoms in their structure. The spectrum of synthesized poly (styrene sulfide) is also consistent with those presented by Aeiyach and Lacaze [16], and in this case the poly (StS) was obtained via anodic or cathodic electrochemical polymerization of **1**.

In the case of IR spectroscopy studies, also good agreement between experimental and calculated data was observed. In the spectral region 400–600 cm^−1^, as DFT calculations show, the line with maximum below 520 cm^−1^ should appear for poly (styrene oligosulfides). The position of this line relates to the length of the oligosulfide bridge, the longer the -S‒S- sequence, the lower is the band position. Also, the other trends observed in experimental results such as increase of low wavenumber shoulder accompanying the line at 700 and a new band at 720 cm^−1^ in IR spectra of poly (styrene oligosulfides) are justified by theoretical calculations. According to DFT calculations in the high frequency region of the IR spectrum, the presence of oligosulfide sequences in poly (styrene oligosulfide) mainchains should result in a discrete structure of bands related to stretching modes of aliphatic CH_2_ and CH groups. This effect is well visible also in the experimental spectra—especially in the case of the poly (StS/S_8_)_1.0_4 sample.

Comparing the FTIR spectra of synthesized polysulfides with the well-known FTIR spectrum of poly (styrene) [41], which constitutes the reference in our work, the CH_2_ stretching modes of poly (St) are found around 2850 cm^−1^ and 2920 cm^−1^, while the bending mode of CH_2_ appears near 1450 cm^−1^. In case of poly (StS) and poly (StS/S_8_) copolymers the bands corresponding to the CH_2_ stretching modes exhibit significantly weaker IR absorptions comparing to the poly (styrene), while the bending mode of CH_2_ is also noticed at around 1450 cm^−1^. The absorption of the lone CH group in the bending mode appears in poly (styrene) in the approximate region of 1250–1380 cm^−1^ [41], particularly at 1375 cm^−1^ in the analyzed poly (styrene), representing low intensity. In the synthesized styrene sulfide copolymers, this band probably is shifted and can be ascribed to the lines with maxima at approximately 1405 cm^−1^ and notably higher intensity. The shift toward higher wavenumbers may be explained by the neighborhood of the sulfur atom(s) in the polysulfide structure. However, we only tentatively suggest, that because this signal also may be attributed to the CH_2_ deformation in the neighborhood of the sulfur atom(s).

Notably, the vibrations corresponding to the aromatic (phenyl) ring in poly (St), poly (StS) as well as in poly (StS/S_8_) copolymers are overlapping and are located at 1490 cm^−1^ as well as nearby 1600 cm^−1^ (carbon stretching vibrations), above 3000 cm^−1^ (C‒H stretching vibrations) as a multiplicity of weak to moderate bands. In case of poly (styrene), the bands found at 750 cm^−1^ and 694 cm^−1^, and slightly shifted to 760 cm^−1^ and 692 cm^−1^ in poly (styrene sulfide) and its copolymers with S_8_ are assigned to the C‒H out of plane vibrations in the phenyl ring [40]. The weak band located nearby 1070 cm^−1^ can be assigned to the skeletal hydrocarbon chain stretching mode in poly (St) [41] as well as in the poly (StS) and poly (StS/S_8_) copolymers.

#### 3.2.4. NMR and ^13^C NMR Spectroscopy Analysis

**Figure 10:**
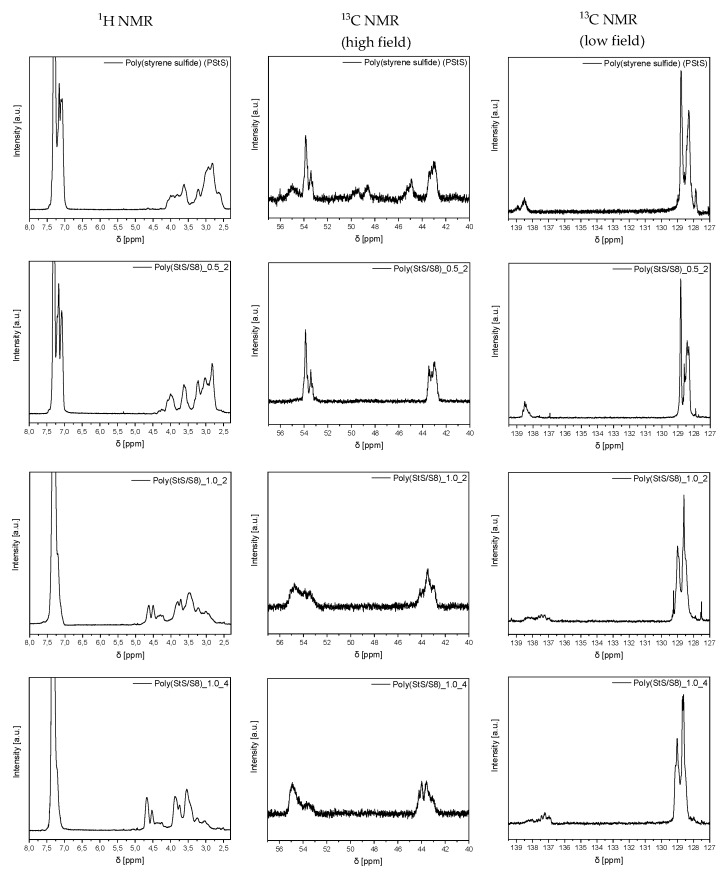
The ^1^H NMR and ^13^C NMR spectra of poly (StS) and poly (StS/S_8_) copolymers.

The proton (^1^H) and carbon (^13^C) NMR spectra of styrene sulfide homopolymer (PStS) as well as its copolymers with sulfur (poly (StS/S_8_) are shown in Figure 10. Although the magnetic resonance spectroscopy measurements are not suitable to supply an information concerning for example the distribution of sulfur atoms in the hydrocarbon mainchain, the NMR analysis constitutes the basic analytical method extensively used to confirm the general structure of polymeric materials or at least to confirm its purity. Hence, the measurements were included in our investigation of poly (StS) and poly (StS/S_8_) copolymers.

The ^1^H NMR as well as the ^13^C NMR spectra of poly (styrene sulfide) have already been registered and extensively analyzed in the past [13,16,42]. In the case of poly (styrene sulfide) obtained via bulk polymerization of **1**, initiated by potassium thiophenolate (PhSK), the region in the high field of the ^1^H NMR spectrum, corresponding to the methylene (CH_2_) and methine (CH) protons is quite complex. The methylene protons give a complex multiplet centered approximately at 2.8 ppm, that is not well-separated from the methine multiplet located at ca. 3.7 ppm. Values obtained herein are slightly shifted comparing to the reference ones [13], in which methylene protons are described as a multiplet centered at ca. 2.55 ppm and another multiplet attributed to the methine protons is centered at ca. 3.3 ppm. The multiplicity of bands observed in the ^1^H NMR spectrum of the synthesized poly (StS) indicates the ambiguous microstructure of poly (StS) and the fact that the regular head-to-tail configuration is probably not the only one that occurs in the poly (StS). This problem was also discussed in Section 3.2.2 in the course of the analysis of the Raman spectrum of poly (StS) and the unexpected presence of a band relating to the S‒S sequence, that should not be present in the structure of the corresponding head-to-tail configuration. Moreover, the aromatic region of the ^1^H NMR spectrum (7.0–7.4 ppm) is characterized by four signals instead of two as reported in reference [13], which may suggest that the phenyl groups are located randomly along the macromolecules and the poly (StS) possess rather atactic than well-organized microstructures.

The measured ^13^C NMR spectrum of poly (StS) is consequently more complex comparing with those previously described [13,16,42]. In the low field region it is easy to distinguish the phenyl (C_arom_) carbon as an ill-resolved peak corresponding to the diversified tacticity of poly (StS), centered at 138.5 ppm (143.4 ppm [13]; 140.5 ppm [16,42]) and layered aromatic (CH_arom_) carbon signals with maxima at 128.8 ppm (meta position), 128.3 ppm (ortho position) and 127.8 ppm (para position), respectively. Excepting shape of the bands, positions of δ as well as the intensity ratio are quite similar to the reported characteristics [16,42]. In the high field region two main signals may be identified. The first with maximum at 43.0 ppm likely related to the mainchain methylene carbon (CH_2_) (40.45 ppm [13]; 37.8 ppm [16]; 37.7 ppm [42]) and the second one found at 53.8 ppm attributed to the mainchain methine carbon (CH) (53.3 ppm [13]; 50.4 ppm [16]; 50.6 ppm [42]). Some additional signals, however, with lower intensity, may be also found in this region and, very likely, should be treated as some artefacts.

**Figure 11:**
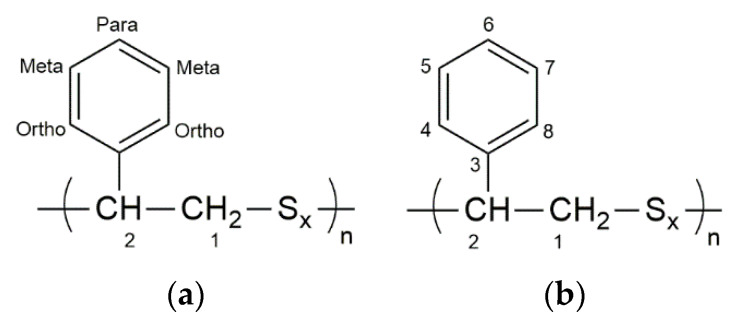
The assignment of numbers to individual atoms in the structure of synthesized polysulfides related to (**a**) ^1^H NMR; (**b**) ^13^C NMR.

The NMR chemical shifts of particular signals in poly (StS) and poly (StS/S_8_) copolymers have been collected in Table 2 and Table 3 according to the atom numbers assigned in Figure 11a (^1^H NMR) and Figure 11b (^13^C NMR). Due to ambiguous and problematic analysis, the values in Table 2 and Table 3 were assigned as values of the maximum of the main signals in the multiplets or presented as the range of δ values.

Following the ^1^H NMR analysis, one can see that the chemical shifts characteristic for CH_2_ and CH protons are relatively similar in the spectra registered for poly (StS/S_8_)_0.5_2 and poly (StS). However, the shape of the signals is different. Notably, in the case of copolymers with increased initial content of sulfur from 0.5 to 1.0 (poly (StS/S_8_)_1.0_2) and poly (StS/S_8_)_1.0_4)), all signals are shifted toward lower field with the new arrangement. The signals are overlapping and form multiplets in the regions of 2.70–4.00 ppm and 4.10–4.80 ppm, which are attributed to the methylene CH_2_ and methine protons CH, respectively.

The effect of extended (co) polymerization time may be discussed based on comparison of the ^1^H NMR spectra of poly (StS/S_8_)_1.0_2 and poly (StS/S_8_)_1.0_4. Longer time of co(polymerization) leads to an increase of the relative intensity of signals centered at 3.50, 3.80 and 4.60 ppm and their slight shifting to lower field. According to Aliyev et al. [43] and Duda [21], such trends result from the longer sequence of sulfide bonds (higher sulfur rank) in the polysulfide structure and it is consistent with the results presented herein.

#### 3.2.5. Thermal Analysis (TGA-DSC)

**Figure 12:**
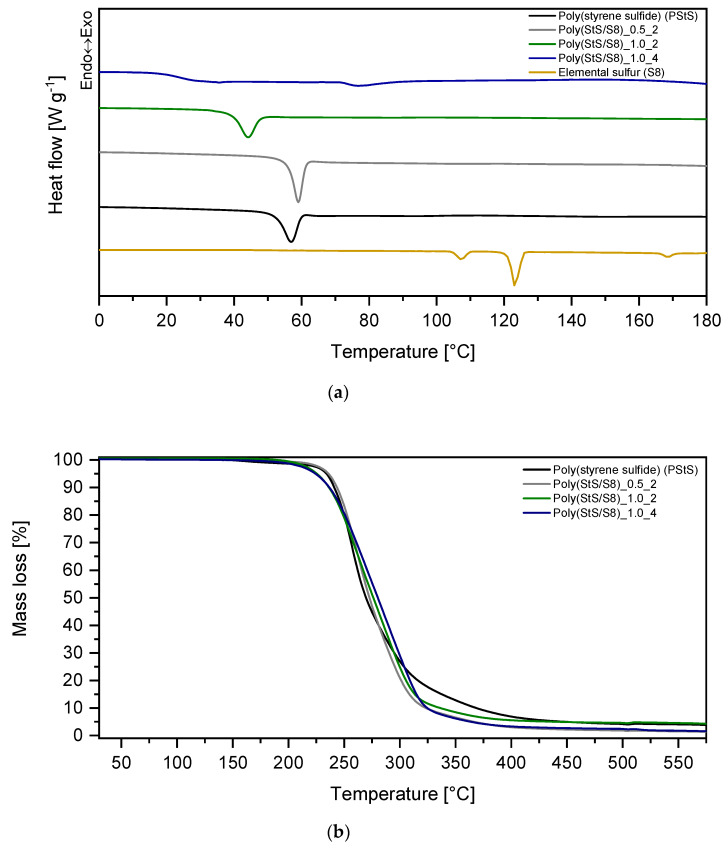

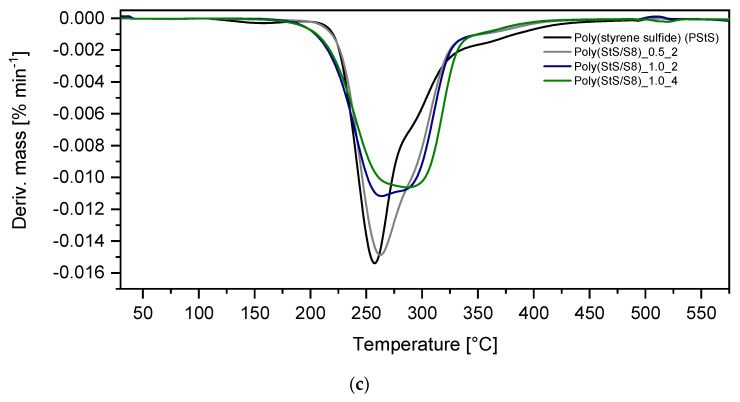
Thermal analysis of synthesized polysulfides (**a**) differential scanning calorimetry (DSC)—first heating scans; (**b**) thermogravimetry (TGA); (**c**) differential thermogravimetry (DTG).

The differential scanning calorimetry measurements (DSC) of the obtained polymeric materials were carried out in order to investigate their thermal stability and range of temperature of phase transitions, mainly the glass transition temperature (T_g_). Moreover, comparison of polysulfides DSC curves with the curve of elemental sulfur provided an additional evidence that the sulfur is chemically bonded in polysulfides structures and no longer exists in a form of a pure, crystalline element (S_8_). The lack of elemental sulfur in the analyzed polysulfides was confirmed in the Raman investigation discussed in Section 3.2.2. The thermal stability of the poly (StS) and poly (StS/S_8_) copolymers was determined. The onset decomposition temperature was established as a temperature required for 5% mass loss during decomposition (T_5%_). The results are presented in Figure 12 and in Table 4.

The DSC curves presented in Figure 12a and the data collected in Table 4 reveal that the glass transition of analyzed polysulfides occurs in the range between 25–58 °C. The homopolymer of styrene sulfide (poly (StS)) and the polysulfide copolymerized for 2 h with S_1 [0]_/StS _[0]_ ratio equal 0.5, exhibit the highest temperature of glass transition, which is very similar for both. The T_g_ is lowering with the increase of initial sulfur content (S_1 [0]_/StS _[0]_ ratio) and consequently higher sulfur rank as well as with the extended copolymerization time. This is consistent with the results obtained by Fitch and Helgeson for the poly (paraxylene polysulfides) and poly (decamethylene polysulfides) [44]. The higher the sulfur rank (the longer sulfide sequence in polysulfide polymer), the lower is T_g_ of the polysulfide [44]. The glass transition temperature of poly (styrene sulfide) is significantly lower than T_g_ of its analogue without sulfur, namely poly (styrene), with T_g_ at 107 °C [45]. However, the poly (StS) as well as the analyzed copolymers occur at room temperature in glassy state or near it in case of poly (StS/S_8_)_1.0_4. The domination of amorphous phase and the lack of crystallinity in poly (StS) and poly (StS/S_8_) copolymers confirm the random arrangement of repeating units of polysulfide macromolecules and consequently provide the atactic microstructure.

In the range of 0–180 °C, melting of potential crystalline phase was not observed. This fact as well as the relaxation effect during glass transition indicate the amorphous nature of synthesized copolymers. The DSC curve of elemental sulfur (S_8_) exhibits three characteristic peaks at around 107 °C, 120 °C and 165 °C, respectively. The first one is related to the transition of orthorhomibc sulfur (Sα) to monoclinic sulfur (S_β_), the second refers to the melting of crystals of monoclinic sulfur, and the last one to the thermally initiated homopolymerization of elemental sulfur. The lack of these three peaks in curves of poly (StS/S_8_) copolymers proves that elemental sulfur (S_8_) no longer exists, as suggested by Raman spectroscopy.

The onset decomposition temperature (T_5%_) is very similar for all synthesized polysulfides, differing barely in 10 °C. In general, polysulfides with a higher amount of sulfur (S_1 [0]_/StS _[0]_ = 1.0) exhibit slightly lower decomposition onset (224–225 °C) comparing with the homopolymer of styrene sulfide (234 °C). The decomposition kinetics of synthesized polysulfides is similar as well, regardless of the time of (co) polymerization and initial amount of sulfur. Copolymers exhibit smooth decomposition indicating its high purity. The TGA investigation provides important information concerning its potential processing and application pathways. It suggests that the careful processing should not be performed above 200 °C that is the requirement for particular processing methods and moreover exclude their application above the mentioned temperature. Recently polysulfides were applied as curing agents for synthetic rubbers [46]. The classic vulcanization temperature usually does not exceed 180 °C and the obtained results may indicate its potential utilization in the field of rubber crosslinking.

## 4. Conclusions

In the present work, the ability of styrene sulfide (2-phenylthiirane) for copolymerization with elemental sulfur (S_8_) below the thermodynamic barrier of sulfur homopolymerization (the floor temperature equal 159 °C) using potassium thiophenolate (PhSK) as an anionic initiator was demonstrated. As a result, new, hitherto unknown copolymers with sulfur (-S_x_-) sequences in the main chain were synthesized. Characterization of the obtained products has led to the following conclusions:

(1) The Raman spectra of poly (StS/S_8_) copolymers constitute a conclusive evidence that elemental sulfur (S_8_) undergoes anionic copolymerization with styrene sulfide (2-phenylthiirane). The lack or low intensity of bands characteristic for cyclooctasulfur (S_8_) and the new diversified bands in the range characteristic for S*‒*S bonds (380–540 cm^−1^) and C*‒*S bonds (700–750 cm^−1^), related to various length of the sulfur bridges in linear polysulfides, are considered as an evidence of a successful copolymerization process.
(2) The lack of melting peaks characteristic for elemental sulfur (S_8_) at polysulfides DSC thermograms constitute additional proof that the sulfur used in these reactions is chemically bonded in a polysulfide structure.
(3) Results of vibrational spectroscopy supported by DFT calculations delivered an information concerning the sulfur rank of the synthesized polysulfides. According to the obtained results, polysulfides formed in the studied processes contain different length of sulfur bridges, with dominant contribution of polymers with sulfur atom sequences longer than 2. The length of sulfur sequences in copolymers is dependent on the initial molar ratio of sulfur to styrene sulfide as well as on the reaction time. The average length of the sulfide bridges varies (increases) with the molar ratio and reaction time.
(4) The thermal investigation indicates that the synthesized polysulfides are stable up to the temperature of approximately 200 °C and may be applied in the future as sulfur donors in the field of rubber crosslinking processes.
(5) This work extends our earlier studies focused on so called ‘sulfur transfer processes’ and sulfur-rich compounds obtained thereby [47,48,49].

## Figures and Tables

**Table 1:** Composition of poly (StS) and StS/S_8_ copolymers as well as the (co) polymerization process conditions.

Sample Symbol	S_1 [0]_/StS _[0]_ Ratio	S_8 [0]_/StS _[0]_ Ratio	Reaction Time (h)	Reaction Temperature (°C)	Color of Crystallized Product
Poly (StS)	0.0	0.000	4.0	95	Pure White
Poly (StS/S_8_)_0.5_2	0.5	0.063	2.0	95	Ivory
Poly (StS/S_8_)_1.0_2	1.0	0.125	2.0	95	Light-Yellow
Poly (StS/S_8_)_1.0_4	1.0	0.125	4.0	95	Light-Yellow

**Table 2:** The ^1^H NMR chemical shifts in poly (StS) and StS/S_8_ copolymers.

Sample Symbol	^1^H NMR δ [ppm]			
	H_1_	H_2_	H_Ortho_	H_Meta-Para_
Poly (StS)	2.5–3.4	3.5–4.2	7.16	7.30
Poly (StS/S_8_)_0.5_2	2.5–3.4	3.4–4.4	7.17	7.30
Poly (StS/S_8_)_1.0_2	2.7–4.0	4.1–4.8	7.20	7.33
Poly (StS/S_8_)_1.0_4	2.8–4.0	4.2–4.8	7.21	7.34

**Table 3:** The ^13^C NMR chemical shifts in poly (StS) and StS/S_8_ copolymers.

Sample Symbol	^13^C NMR δ [ppm]					
	C_1_	C_2_	C_3_	C_4_, C_8_	C_5_, C_7_	C_6_
Poly (StS)	42.99	53.87	138.51	128.30	128.79	127.87
Poly (StS/S_8_)_0.5_2	42.97	53.83	138.45	128.53	128.77	128.36
Poly (StS/S_8_)_1.0_2	43.53	54.75	137.47	128.93	128.99	128.60
Poly (StS/S_8_)_1.0_4	43.58	54.90	137.24	129.02	129.12	128.62

**Table 4:** Glass transition temperature and thermal stability of poly (StS) and poly (StS/S_8_) copolymers.

Sample Symbol	T_g_^1^ [°C]	T_5%_^2^ [°C]
Poly (StS)	55.1	234
Poly (StS/S_8_)_0.5_2	58.0	235
Poly (StS/S_8_)_1.0_2	42.5	225
Poly (StS/S_8_)_1.0_4	25.5	224

^1^ Glass transition temperature; ^2^ Decomposition temperature at 5% mass change.
